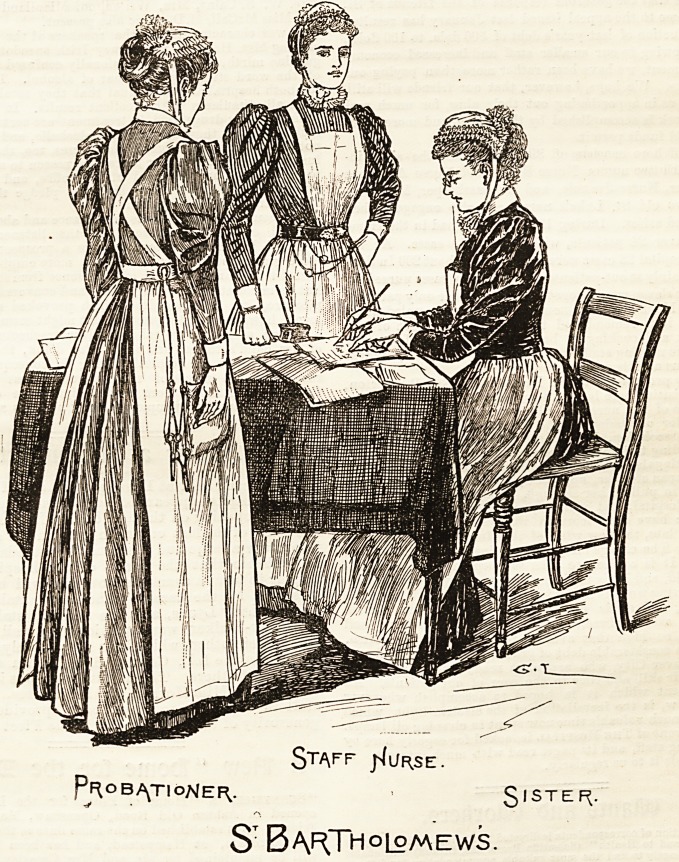# The Hospital Nursing Supplement

**Published:** 1896-02-22

**Authors:** 


### "The Hospital" Nursing Mirror.

Being the Extra Nursing Supplement of "The Hospital" Newspaper.

[Contributions for this Supplement should be addressed to the Editor, The Hospital, 428, Strand, London, W.C., and should have the word "Nursing" plainly written in left-hand top corner of the envelope.]

### News from the Nursing World.

### THE PATIENTS' TREAT.

The entertainment annually given to the patients at the Royal Hospital for Children and Women in Waterloo Bridge Road could not take place at Christmas this year in consequence of some cases of mumps occurring at that season in the wards. The hospital's modest proportions not facilitating satisfactory isolation, it was decided to postpone the treat until later on. Accordingly on February 12th a very pleasant gathering of friends took place in one of the wards, in which the majority of the in-patients were collected. Some excellent vocal and instrumental music was provided by various friends, and the patients had the additional and substantial pleasure of receiving many welcome gifts, which they evidently considered quite as acceptable, through the medium of an ingeniously designed large red "post office," as if they had been distributed six weeks earlier from a gorgeous Christmas tree.

### THE WRECK OF AN AMBULANCE.

A nurse proceeding from the Western Fever Hospital to the hospital at Winchmore Hill with an ambulance load of convalescent scarlet fever patients had a curious experience on Valentine's Day. According to the Press report, the ambulance had a prosperous career from Fulham to the North-West London Hospital, where it stopped, eventually proceeding towards Highgate. In turning off to Holly Village the wheels "skidded" on the tram rails, an axle snapped, and the body of the ambulance fell to the ground, causing considerable alarm to the nurse and her party of eleven convalescents. Luckily, no one was hurt, and the boys and girls doubtless resigned themselves philosophically to their rather long detention in their confined quarters, as, of course, it was out of the question for such infectious young people to leave the vehicle until the fresh ambulance, for which the driver telegraphed, arrived on the scene.

### KENT AND CANTERBURY NURSING INSTITUTE.

This institute chronicles a satisfactory year's work for 1895. The report which was adopted at the recent annual meeting showed that the earnings of the nurses had increased from £1,193 in 1894 to £1,329. The great event of the past year has been the removal of the institute from St. Margaret Street to new and better quarters, which was accomplished in April, a good house and garden having been purchased in Burgate for £1,000. Of this sum the large balance at the disposal of the committee at the end of last year enabled £250 to be at once paid, and it is confidently hoped that the mortgage upon which the remainder was raised will be paid off in the course of a few years. Separate bed-rooms for thirteen nurses are provided, and four sitting-rooms, while the possession of a good garden has been a great boon to all the staff. The committee encourage their nurses to join the Royal National Pension Fund by paying a portion of their premiums, which portion it is intended, as soon as finanees permit, to increase, so as to offer every inducement to the nurses to make adequate provision for old age or sickness. Several of the nurses who have not yet availed themselves of these advantages are intending to do so during the present year. The salaries of the lady superintendent and the nurses have been raised, and the staff slightly increased, so that the Kent and Canterbury Nurses' Institute may certainly be congratulated upon its well-earned prosperity. While the institute itself is fully self-supporting, the district work depends on subscriptions and donations, which have not met expenses by a few pounds the last two years. It must be hoped that this small debt may speedily be cleared off, and increased funds assured for this year, that there may be no curtailment of this important branch of the nursing work.

### PRIVATE NURSES AT LEEDS.

The twentieth annual meeting of the Leeds Trained Nurses' Institution has been held, and the staff now numbers 95 private nurses and 15 probationers undergoing hospital training. The sum of £100 has again been handed to the Trust Fund established to secure to each nurse an additional £10 per annum when she enters on her pension from the Royal National Pension Fund for Nurses.

### A SHARE IN A ROOM.

By the provision of a trained nurse for night duty the Kilrush Guardians show themselves considerate for the infirmary patients under their charge; but they have hitherto failed to realise that their responsibilities do not end with the sick poor. For the nuns who look after the patients during the day the head of their Order is responsible, but the solitary trained nurse is, according to a medical contemporary, very badly lodged. Although a room in the infirmary can be spared for a paying patient, the only apartment for the night nurse is reported to be also "the only one available as a casual ward, and is at times used as such!"

### QUEEN'S NURSES AT INVERNESS.

An amateur dramatic and musical entertainment was held the other day in aid of the Inverness branch of the Queen's Jubilee Institute. The funds are badly in need of increased support, a second nurse being urgently demanded for the sick poor of the district. The performances were attended by crowded audiences, and were held under distinguished patronage, but we learn with regret that a very small sum remained for the charity after all expenses had been paid.

### THE HOSPITAL SHIP "COROMANDEL."

In the daily reports from Ashanti the attention of nurses is almost universally centred on the admirable work of the Medical Staff Corps, and on the accompanying items of news from the well-equipped emergency hospitals. Prevention of disease is one of the Medical Staff Corps' primary duties, and appears to receive the attention which its importance demands. The cases admitted to the hospitals are chiefly medical, and form a practical endorsement of the advice we are so often called upon to offer to nurses with regard to "army nursing." Ladies who fill honourable posts as Her Majesty's nursing sisters are aware that disease is far more in evidence than the wounds which people at home are apt to account the chief horror of war. On the beautiful hospital ship "Coromandel," stationed off Cape Coast Castle, every facility is offered for the medical treatment and intelligent nursing of the sick. The cheerful, airy wards are discarded by all patients who can be moved on deck, where easy chairs and lounges are provided, and meals served to the men who remain all day in the open air. Whatever other lesson the Ashanti expedition may teach, it has already shown with what extreme efficiency modern science can deal with unhealthy surroundings, and with what satisfactory results modern nursing can be supplied to the soldier of to-day.

### NURSES FOR LEPERS.

Before "joining" a leper settlement, it is necessary to learn first if an English woman would be admitted, in what capacity, and whether any remuneration is offered. There is a great deal of attention and care needed by many lepers who require no actual nursing at all although this is frequently provided by natives of the country wherein the hospital is situated, who speak the patients' language. Certainly no English nurse should attempt to go out without obtaining full particulars from the resident medical superintendent, who would at once inform her whether her services are required. In The Hospital of May 27th, 1893, there was a charming account of the Colombo Leper Hospital, and from time to time we have mentioned those in Norway, New South Wales, at Robben Island (South Africa), Trinidad, and other islands, as well as districts in India where leprosy is common. Nurses who especially desire to nurse lepers are therefore advised to apply to the managers of hospitals already in existence, or to advertise in our columns for the kind of appointment they aspire to fill.

### HOSPITAL FOR SICK CHILDREN, BRIGHTON.

New sleeping accommodation is to be provided for the nursing staff at the Alexandra Hospital for Sick Children at Brighton. This step was proposed and unanimously agreed to at the recent annual meeting, Mr. Dewé, who introduced the proposal, remarking that "the nurses were devoted to their work, and the least they could do was to give them proper sleeping accommodation when their work was done"—a sentence which, it is pleasant to note, was received with "applause." The new building is to be in the grounds connected with the hospital proper by a covered way, and will provide for twelve nurses. We trust the committee intend providing entirely separate rooms for the nurses, and those not divided by mere matchboard partitions, for that plan alone can secure for a nurse the unbroken rest which she needs to fit her for her daily work. Plans have been prepared at an estimated cost of £1,200, and contributions towards this sum have already been received amounting to £671. The committee have been empowered to devote a sum of not exceeding £800 Consols to the building if required.

### ENTHUSIASTIC BUT PORTIONLESS WORKERS.

Many of the queries addressed to us on the subject of nursing abroad contain a stipulation that the writer must have her expenses paid, as she has no private means. To all such correspondents we invariably give deterrent advice, for it is obvious that nurses without means are better in England, where they are within reach of their friends in case of illness or enforced idleness. Unless a nurse goes out on a definite agreement to a well-established colonial hospital, she is not likely to get a free passage, much less an outfit. The time is past when a brief period of training was sufficient equipment for a woman intending to take up nursing abroad. Qualifications are more carefully sifted nowadays, and the demand, even for fully-trained nurses, is comparatively limited, since many hospitals have organised nursing schools of their own in all parts of the world. For any nurse without means to go to a colony in the hope of immediately making a good living is a mistake for which she is likely to pay dearly. She may have to wait long for work, and she will find living costly, and will, moreover, receive a sorry welcome from colonists, wholly or partially trained, who instinctively dislike and are jealous of the stranger who expects to supersede them.

### HOSPITAL SKETCHES.

Miss Gethen (formerly "Sister Queen" at the London Hospital), whose recitals of her own original stories of hospital life have become deservedly popular in many quarters of late, is prepared to undertake engagements for public or drawing-room entertainments. For terms apply to "Secretary," 52, St. John's Wood Road, N.W. Miss Gethen's sketches invariably please because they are so evidently drawn from life, and her way of telling them is just what it should be.

### SHORT ITEMS.

The Dover Trained Nurses' Institution has just issued its twenty-first annual report, and gives an excellent record of the satisfactory work done by the staff.—It is stated that the Bridgwater Guardians consider that the recommendations of the Local Government Board as regards a night nurse are reasonable, but the offer of £15 per annum as salary will hardly tempt a competent trained woman to apply for the post.—The Leicester Guardians have appointed Miss Masters as midwifery nurse at a salary of £65 a year.—The Wootton District Nursing Society has issued a most satisfactory annual report.—The "Transvaal Supplement" of *Black and White* for February 15th contains a sketch of the General Hospital, Johannesburg, which will interest the many home friends of the English nurses who so recently took up their work within its walls, and who arrived in South Africa in such exciting times.—Out of thirty-eight nurses who went up for examination at the Prince Alfred Hospital, Sidney, last month, thirty-four passed most creditably, the examiners saying that they had never before received so many excellent answers.—Sweden now possesses three women doctors, Fröken Maria Folkeson, the daughter of a member of the Swedish Parliament, having just taken her degree of Licentiate of Medicine at the Caroline Institute, Stockholm.—Miss Clara Barton, President of the American National Red Cross Society, who has organised an expedition for distributing relief to the Armenians, is reported to have reached Constantinople.

### Lectures on Nursing.

By a Superintendent of Nurses.

### X.—DIET. RECIPES. DIABETES.

It is very necessary that the diet of sick and convalescent patients should be varied as much as possible, for the most dainty food soon palls upon the taste if presented too frequently. Beef tea used to hold a very high place as an article of diet, but now much more importance is attached to milk. This, if taken hot, is more digestible if not allowed quite to boil. It makes a most refreshing drink if soda water is added to the hot milk.

Eggs are exceedingly nutritious, containing a large quantity of oil in their yolks. They should not be boiled, but put into boiling water and allowed to remain in a covered saucepan on the hob for five minutes. The albuminous (white) portion will be solid, but much lighter and more creamy than if allowed to boil. In poaching an egg it should be taken out of its shell and put into a saucepan of water that has been boiling for a minute or two, and allowed to stand on the hob for five minutes as in the former case. There is another way of taking an egg, which I can strongly recommend. Squeeze the juice of half (or the whole) of a lemon into a tumbler three parts full of hot or cold water, sweeten to taste, and add an egg thoroughly well beaten. It is much more easily digested than when put into milk, and is a very sustaining and pleasant drink. If taken cold, ice can be added, making it most refreshing.

Melt tea is highly nourishing, but is not very generally known. It is made in the following way: Take a freshly-killed sheep's melt; chop it into small pieces, put into a pint jar of water with a little salt. Cover the jar, and let it stand by the side of the fire. Bring the temperature to near boiling point. Let it simmer for eight hours, flavour with a brown onion and little pepper. Strain it and serve.

An excellent veal broth is made as follows: Take a knuckle of veal chopped up like a cucumber; put it in a jar with a pint of water. Cover it up and let it simmer on the hob for eight hours, and add some Valentine's juice to flavour it.

To make strong beef tea take a pound of top side of beef, strip it of all gristle, skin, and fat, cut it up into small pieces and put it into a stone jar with about three-quarters of a pint of water; cover it and let it stand for an hour, then place the jar in a large saucepan of boiling water, and let it remain for three hours, if possible, on the hob. Skim it when it is cold, and heat as required, adding salt after it is boiled. Sometimes a patient will like it much better if a lump of ice is added and it is given quite cold.

In some cases where the digestive powers are much impaired it is necessary to partially predigest the food before feeding the patient with it. This can be most conveniently done by using Benger's Liquor Pancreaticus. A teaspoonful of the liquor, together with a pinch of bicarbonate of soda, should be added to the milk or other food, and then the whole gently warmed for about twenty minutes. The object of warming like this is to facilitate the action of the pancreatic ferments, which are most active at a temperature of 140 deg. At the end of twenty minutes, when the necessary changes have taken place, the milk must be raised nearly, if not quite, to boiling point, in order to stop any further action of the pancreatic ferment. If these are allowed to go on acting, the milk becomes bitter and nauseous. Where the patient has a great dislike to even a suspicion of bitter flavour, it is better to prepare food fresh each time.

Whey can frequently be taken by persons who have excessively weak stomachs. To a pint of milk slightly warmed add a large dessertspoonful of liquid rennet; let it stand for six hours, then put it into a strainer; breaking the junket into small pieces let the liquid drip into a basin or jug.

Sweetbreads, if properly prepared and cooked, are most nutritious and easily digested. They should be soaked in lukewarm water, to which a teaspoonful of vinegar has been added, for two hours, changing the water once or twice during the time. Then they should be thrown into boiling water, and allowed to simmer for five or ten minutes till they are firm and round, but not hard. After this they should again be put into cold water for about ten minutes, taken up again, wiped dry, and set aside till quite cold, when they are ready for cooking. Stewed sweetbreads are often preferred by sick people, and the recipe for cooking them can be found in any cookery book.

Boiled chicken minced in a mincing machine, with a small quantity of cream mixed with it, is a delicate article of diet, and can be eaten without much trouble.

Food should never be kept in the sick room; beef tea and other liquid foods can be easily warmed in an adjoining room by using a spirit lamp.

Tea made with boiling milk is sometimes recommended as nourishing and stimulating, but more tea will be required than if made with water.

In the treatment of some maladies, much depends on the proper course of dieting pursued. Diabetes is one. It is a disease chiefly characterised by the presence of a large quantity of sugar in the urine. The patient suffers greatly from thirst and hunger, whilst the body wastes away rapidly. The sugars in the system are used up as a rule in the circulation and in a state of health are not found in any appreciable quantity in any of the excretions. Diabetes is usually brought on by injury or disease of the brain and mental excitement or worry. Any great "strain" of continued anxiety may cause it. Sometimes errors of diet; an excessive use of sugar, for example, may bring it on.

The urine is usually of a high specific gravity, perhaps 1,040 deg.; the skin is dry and harsh, the tongue red or glazed, or slightly furred; the mouth dry and clammy; the breath sweet like hay; the lips and gums covered with sticky mucus, and the face wears an expression of weariness. Various organs are more or less diseased—brain, liver, pancreas, lungs, and kidneys. Men are more frequently attacked than women. Middle-aged people are most subject to it, and are more likely to recover if they are from forty-five to fifty years age. They do recover to a certain extent frequently if under judicious treatment, but much depends upon the food they take. Usually a diet is prescribed containing the smallest possible quantity of sugar or starch, which is, of course, easily turned into sugar.

Naturally, the patient longs for ordinary bread, which is, as a rule, forbidden, and he is often ordered instead gluten bread, which is made of flour out of which all the sugar is extracted. Bran bread and almond cakes are also given. It is the nurse's duty to see that the doctor's injunctions are strictly carried out. In making a diet scale it is very important to give the patient as great a variety as possible. Meat is generally looked upon as the chief constituent. Some vegetables are permitted, such as the green parts of asparagus and celery, cabbage, spinach, broccoli, Brussels sprouts, mustard and cress, and young French beans. Cheese may be eaten as well as cream and butter. Cold tea with some lemon juice squeezed in will be found not only a very refreshing drink, but will cleanse the mouth. Buttermilk is sometimes allowed, and this is lighter than milk and very nourishing. Sucking ice and washing the mouth with iced water will do much towards assuaging the thirst. All sweet drinks are prohibited, also all cereals, peas, potatoes, parsnips, carrots, and beetroot.

The first sign of improvement is a moist skin and an abatement of thirst; less urine is passed, and it contains less sugar; the specific gravity also being lessened, and the patient gains weight. If the disease does not yield to treatment the symptoms usually become aggravated, the sight becomes dim, complications take place, pneumonia comes on, and the patient sinks under diabetic coma or by gradual exhaustion. A person in health, as a rule, passes about fifty ounces of urine in twenty-four hours, but if suffering from diabetes may pass as much as forty pints in the same time, and it will be the nurse's duty to measure this daily, and to report the quantity, the specific gravity, and the amount of sugar. She will also have to weigh the patient week by week. People suffering from this complaint should always avoid wet and cold, and wear flannel underclothing, which should be frequently changed.

When the amount of sugar has to be estimated daily, perhaps the simplest method of doing so is by yeast. Some of the urine taken from the total amount collected in the twenty-four hours is placed in a specimen glass, its specific gravity is taken and noted, and a piece of German yeast about the size of a pigeon's egg is placed in it; the height of the urine in the glass should then be marked on the outside with pen and ink. The urine is then set aside for 24 hours. The glass must then be filled up to the mark with water in order to replace what will have been lost by evaporation. If the specific gravity is then again taken, the number of grains of sugar per ounce will be represented by the difference between this specific gravity and that determined on the previous day. For instance, if on the first day the specific gravity was 1,040, and on the second 1,025, the number of grains of sugar per ounce equals 1,040—1,025, *i.e.*, 15 grains. It is then only necessary to multiply the number of grains per ounce by the number of ounces of urine passed in the 24 hours, in order to determine the number of grains passed in the 24 hours. Thus, in the above case, if the patient were passing 100 ounces of urine per diem, then $15 \times 100 = 1{,}500$ would be the number of grains passed in the 24 hours.

### Private Nursing.

By Sister Grace.

### II.—PRIVATE NURSES PAST AND PRESENT.

We pride ourselves, and justly so, on the disappearance from our midst of Sarah Gamp, with her accompaniments of brandy bottle and ready-prepared mourning; indeed, when one looks back to what nursing was thirty years ago, one must feel that no branch of industry has made greater progress in the same time. The matron under whom I worked was the first Nightingale Sister at St. Thomas's, and she told me many amusing things about the nurses who were there when she joined them as a probationer. Private nursing was then in its infancy, and when she became matron of ——— Hospital, twenty-five years since, she began to organise a nursing home for private nurses. At first, and, indeed, for many years, the difficulties seemed almost insurmountable. The nurses in the hospital were few in number, untrustworthy in many ways, and not all of them free from Mrs. Gamp's besetting temptation. One reason of the extremely unsatisfactory stamp of nurse employed at that time in all hospitals was that nursing was considered somewhat in the light of a work of expiation, and peculiarly suited to women who had slipped from the straight path into devious bye-ways of dishonesty, inebriety, etc. This made women of a superior stamp somewhat shy of entering the work. Twenty-five years ago the tide was fast turning: Miss Nightingale's noble efforts in the Crimea, and Charles Dickens' uncompromising description of nursing as it was, did much to show how great was the need for true-hearted women to enter upon this difficult field of labour. Our matron began by advertising, and so unpopular was the work that she had the greatest difficulty in getting sufficient nurses to work the wards, far less to start a private staff. Some of her early experiences would be very amusing, only one is so saddened to think what poor invalids endured in those days.

Many times she was tempted to give up the struggle, but, being a high-souled, noble-spirited woman, she would not yield to despair, and through many disheartening failures and disappointments at last succeeded in establishing a private nursing staff that would, I think, be difficult to surpass in general usefulness, high principle, and conscientiousness.

There has been much discussion lately on the subject of the poor pay of nurses. It is certainly not an employment that can be called well paid; still, one must remember that board, lodging, and uniform, added to a salary of from £25 to £40 yearly, is not poor pay for the class of women who pass into the ranks of the nursing profession. Many advocate private nurses having a percentage on their earnings. This system does not commend itself to me, because it seems to lower the work to the level of a commercial transaction, and opens a wide door to much that is objectionable where nurses are not conscientious. I hope and believe that the number of these is small, but it would be absurd to suppose that among so many thousands there are not some who fall very short of perfection. I think our plan was better—to give a good salary, and to encourage them to save by giving a bonus added to their own savings. They began with £24 and increased to £34, when, their bonus being £6, their money for the year came to £40, and uniform added to the value of about £6.

### Position of the Nurse in the House.

We have, indeed, greatly changed for the better since Sarah Gamp and Betsy Prig settled down to supper, having snatched the pillow from under their dying patient to accommodate themselves. All this is gone into the long dead past, but bear with me a few moments, whilst I point out one fault that some nurses (excellent in all other ways) share with those ladies of undying reputation. I allude to the reprehensible practice of talking of their "cases." The more interest a nurse takes in her patients and her work generally the more likely she is to fall into this error. Remember, that you know many things about your patient and the family with whom you are placed that they would be grievously hurt to think you had mentioned outside the house. Your position is somewhat that of clergymen and family lawyers: you know and hear much that should never pass your lips. To speak of these things is a breach of confidence, to say nothing of showing a serious lack of delicacy and kindness. A private nurse needs an exceptional amount of tact (a rare and precious quality). She has many to please; first the doctor and the patient, then, more difficult still, the patient's friends, and lastly the servants. Many nurses, desirable in all other respects, have proved anything but a blessing in the house, on account of their tendency to constant disagreements with the servants. I think the exhortation to "love yourself last" should be constantly in a nurse's mind, and thus she will avoid many pitfalls.

### St. Thomas's Hospital.

The Duchess of Connaught intends accompanying the Duke of Connaught on Friday, when His Royal Highness is to reopen the closed wards at St. Thomas's Hospital.

### Dress and Uniforms.

BY A MATRON AND SUPERINTENDENT OF NURSES.

### ST. BARTHOLOMEW'S HOSPITAL.

FEW costumes are prettier than those worn by the nursing staff of St. Bartholomew's Hospital. Light blue linen of a peculiarly bright hue has, in compliance with modern sanitary ideas, replaced the rich purple-hued merino that until a year or two ago distinguished the sisters of this time-honoured institution. The skirt and bodice, which are quite plain, are united by a band at the waist, the latter buttoning in front. The skirt, which is of ample dimensions, clears the ground with a substantial hem all round. Large linen aprons with square bibs, which button at each corner on to the front of the dress with a small shirt button, are worn over this, and plain turn down-collars and cuffs. The cap is a small Marie Stuart shape, and is edged with two rows of gophered lace about an inch wide, and has cambric strings trimmed to match, which tie in a neat bow under the chin. The staff nurses wear narrow blue and white striped galatea, which from the fact of its being clean and neat is always becoming to the wearer. Their aprons differ from the sisters' in having straps attached to the bibs, which cross behind and are fastened on to the band of the apron with buttons. The cap is the Marie Stuart shape, but is trimmed with Coventry frilling instead of lace like the sisters'. It is gophered, and a thread run through each row, which is drawn in sufficiently tight to shape the cap to the head. Those nurses who have passed their final examination after completing the three years' course are distinguished by a navy blue belt; and the "blue belts," as they are termed, are an important element in the hospital. Dark blue and white twilled galatea is worn by the probationers, and their caps

STAFF NURSE.

PROBATIONER.

SISTER.

ST BARTHOLOMEW'S.

and aprons are of the same description as those of the staff nurses. The outdoor uniform is very neat and simple, consisting of a plain black circular, or a Russian cloak, and a "Princess" bonnet, trimmed with black velvet, a white border, and narrow cambric strings tied in a small bow in front.

### St. Luke's Home, Vancouver.

From a Correspondent.

It is pleasant to be able to begin the present year with the knowledge that the generous response of the friends of St. Luke's Home to the appeal issued last January has resulted in the reduction of last year's debt of 500 dols. to 100 dols., and that, owing to our smaller staff and increased economy in management, we have been rather more than paying our way of late. We hope, however, that our friends will still remember us in apportioning out their alms, for much real charity work is accomplished by the Home, and more could be done did funds permit.

Our staff here consists of Sister Frances, the sister-in-charge, with two nurses, Nurse Hester and Nurse Amy, one probationer, Nurse Jeannie, and a housekeeper, Miss Roycraft. Two old St. Luke's nurses are also engaged when special need arises. During 1895 we have had in the Home in Vancouver 24 patients, with 16 outside cases. At the Lytton Hospital 28 cases were treated, and about 200 Indians treated, mainly as out-patients. Of the forty cases nursed in Vancouver eleven were altogether free, and four only partially paid for. Much serious illness has had to be grappled with, and when it is remembered that the greater part of the housework and cooking at both Vancouver and Lytton is done by the nursing staff, it will be seen that last year was by no means an idle one.

An early probationer at St. Luke's, Miss Eva Pemberton, has been visiting here lately, now being at Lytton seeing the Indian side of the nursing. She was the first Canadian girl to apply for admission to the Metropolitan and National Nursing Association, Bloomsbury, for district work, and is now intending to join at Edinburgh as a Queen's nurse.

A Nightingale nurse was brought to St. Luke's last summer from a Japan steamer, apparently at death's door, but we succeeded in pulling her through. She had been in charge of Peek Hospital, had married, and was on her homeward way. We have done more in the way of accommodating visitors of late, the new wing of the Home being kept apart for them. The charge for people coming and going is 1 dol. per day. It is a pleasure thus to keep in touch with the outer world, and leads to the making of many kind friends.

The nursing work is likely to increase. Dr. Bell Irving is here still, and sends us many patients; but, in British Columbia, as elsewhere, nursing is getting overdone, and prices are so high that average people cannot pay them. We owe a considerable debt of gratitude to the medical men of Vancouver City, who are most ready and unselfish in giving their skill and time to the care of our patients. An improvement which it is hoped to accomplish whenever funds allow, is the installation of the electric light, which will save much valuable time now spent in cleaning oil lamps.

The advent of The Hospital is looked for eagerly here by the nursing staff, and its pages read with much interest. A friend sends it to us regularly.

### Wants and Workers.

[The attention of correspondents is directed to the fact that "Helps in Sickness and to Health" (Scientific Press, 428, Strand) will enable them promptly to find the most suitable accommodation for difficult or special cases.]

Any nurse who may care for a quiet rest in a pleasant little house in a beautiful part of Cheshire, at inclusive terms 6s. 6d. weekly, or 6s. each to two nurses willing to share one room, can apply for particulars, by letter only, to Nurse Mary, 11, Clarence Road, Teddington, Middlesex.

Nursing Abroad.—A correspondent is anxious for some particulars about private nursing in Gibraltar and in Cairo, and to know whether a venture in either place would be likely to meet with success, and what special qualifications a nurse should hold. Will any nurse who has had practical experience of nursing in these two places kindly supply any information on these points she may possess?

### Temperance for Nurses.

The fine room at Grosvenor House which the Duke of Westminster so frequently places at the disposal of charitable associations was very full last week on the occasion of the meeting of the Women's Total Abstinence Union. A number of nurses in uniform were noticeable amongst the audience, and for them the address by Sir Benjamin Ward Richardson was specially intended. In his absence, on account of illness, his speech was read for him by Mrs. Docwra. Lady Elizabeth Biddulph was in the chair, and Mrs. Milligan, Mrs. Finlay, Mrs. Septimus Buss, Miss Docwra, Miss Orme, Mrs. Terrill, Mrs. Servante, Mr. and Mrs. W. S. Caine, Mrs. Wilson, Miss Holland (secretary), and Miss McCall, M.D., were also present.

It was characteristic of the speakers at the meeting, including Mrs. Hawkes, whose racy Irish anecdotes provoked genuine mirth, that they occasionally confused the meaning of the word stimulant with that of alcohol. They claimed for both hospital and individual that they totally abstained from all stimulants with excellent results. In the case of patients many drugs used in treatment are certainly stimulants, although they may not be alcoholic, and this should not be ignored. As regards the nurses, too, the same confusion of terms existed, for there is no reason to suppose that these workers are deprived of tea, coffee, and many other stimulants, although they choose to pledge themselves to abstain from beer and spirits.

The confusion of the terms temperance and abstinence also aroused comment from some of the listeners, and the Women's Union would seem to have a prospect of gaining more general popularity if it placed more emphasis on universal temperance, not only on abstinence from strong drinks, but on moderation in eating, dress, and conversation—work, pleasure, &c. The anecdote which provoked most amusement, however unfitting amusement might seem, referred to a woman said to have taken a silver tracheotomy tube out of her child's throat, and sold it for drink, the child, who was a patient in hospital, dying in consequence of its removal. The name of the institution was withheld, so the amazed listeners were left in ignorance of the place where such inefficient super vision of patients and friends is asserted to exist.

### Bazaar at Devonport.

The bazaar held at Devonport in aid of the Royal Albert Hospital appears to have been very successful from the opening on the first day by Lady Robartes to the close of the last entertainment on the second day. There was a large and fashionable attendance, and it is probable that the funds of the hospital, which appear to be at a somewhat low ebb, will benefit considerably by the bazaar. The naval stall, presided over by Lady Lyons, and the military stall by Lady Forestier-Walker, were particularly well furnished, and so were others which bore respectively the names Royal Marine L.I., County Stall, Town and Utility Stalls, and others. The hospital stall was in charge of the matron, Miss Horner, and her nurses. The naval stall is reported to have been provided by the generosity of the men serving in the Fleet Reserve.

### A New "Home for the Dying."

"Sunnyside," a "Home of Peace for the Dying," was opened in Ashton Old Road, Openshaw, Manchester, on Friday. It is established on the same lines as the well-known "Friedenheim," at Hampstead, and has been founded and will be maintained by Mr. and Mrs. Crossley, of Bowden, who are well known in and around Manchester for their kindness to the sick and poor. The home contains ten beds distributed through four wards, and has been fitted up with every comfort. Besides the wards, there is adequate provision of bed-rooms and sitting-rooms for the nursing staff. Nurse Mary Trotter has been appointed matron, with two nurses—one for day and one for night duty—to work under her. Nurse Trotter is a Queen's Nurse and a member of the Pension Fund, having received her badge at Marlborough House last summer.

### A Book and its Story.

### A REMARKABLE WOMAN.

WHEN the Duchessa di Cajanello was writing this memoir of Sonia Kovalevsky, it chanced that the authoress met Henrik Ibsen one day in Christiania.

"Is it a biography in the true sense of the word, or a poetic image, you are going to give?" the dramatist asked.

"It is her own poem about herself, seen with my eyes, which I mean to interpret," was the answer.

"Quite right," said Ibsen, with a fine touch of irony. "The subject needs to be treated poetically."

And who was "Sonia"? One asks the question with a sense of shame, for to posterity her name should be universally handed down as that of one of the most brilliant and distinguished mathematicians who has ever appeared in woman's form. Sonia Kovalevsky's personality was of a twofold kind. As a genius of no mean order, she was known to the world otherwise than through her present biography; but Sonia Kovalevsky—the woman—has only been revealed through the Duchessa's pen, and the life story which is here unfolded has its own peculiar interest, as the record of an unique personality.

We are introduced to Sonia at the age of seventeen, when she is living with her parents, people who are moving in the upper middle class circles of St. Petersburg society. The household is a commonplace one, where the daughters are expected to be content with the comforts of their home and comply with the ordinary usages of society. But, unfortunately, at that time (1860) certain young women among the upper classes in Russia were in a state of revolt against the established order of things. They had come to the conclusion that domestic life was inconsistent with "mental liberty," "development," "knowledge and light," and that in foreign universities alone could their "cravings" for these blessings be duly satisfied. Their parents, after the manner of parents, were not in sympathy with such aspirations, and met the daughters' ardour with certain common-sense objections. But to Sonia, her sister Aniuta, and their companions, such objections were not to be considered, and they discovered what the Duchessa di Cajanello, with a wholesome accuracy, calls "a very peculiar and characteristic way out of the difficulty." In order to escape the parental authority, marriages were to be contracted with young men who shared their views. These marriages were marriages only in name, the bridegroom going through the ceremony and leaving his wife free and alone. And we are told "this kind of union was becoming so popular among the friends with whom Sonia and her sister associated in the Russian capital that it came to be looked upon as the ideal form of married life." Had the subject of this memoir escaped the influence of these ideas, her career would have been a happier one; but into the vortex she was slowly and surely drawn, and the story of her life is the sadder through it.

Perhaps at first we sympathise with her, for even at an early age Sonia had displayed mental powers of no ordinary kind, and an aptitude for those scientific pursuits in which she afterwards distinguished herself. And so, one day, obeying the teaching of the "New Ideas," Sonia and her sister Aniuta, and another, resolved, in their turn, to look out for some man with whom "an ideal marriage" was to be contracted, and through which means their longed-for freedom could be purchased. Their first advances were made to a young professor, who received them with politeness, but who bowed them to the door.

About fifteen years later, when Sonia Kovalevsky stood at the height of her fame, she one day, at a party in St. Petersburg, met this man, and they joked together about the unsuccessful proposal. They were, however, not to be daunted; and in a short time the step was taken which changed the current of Sonia's life. This time the gentleman on whom they called was a student, Waldemar Kovalevsky, to whom Aniuta offered herself. With perfect frankness he declined the proposal, but said "he would take her sister." This coming to her father's ears, he thought fit to raise objections. Sonia, to whose character thwarting but acted as an incitement, resolved to carry out her plan by eloping. The two contracting parties to this curious arrangement were married in October, 1868; and the marriage turned out as might have been expected. The young couple went to study at Heidelberg. Kovalevsky was clever, industrial, and frugal. He gave himself up entirely to his studies. Sonia's brilliant abilities took her teachers at the German university by storm, but she was not satisfied; and, though in a sense attached to each other, the husband and wife gradually became estranged. Life with Sonia must have had its attractions, but it also had its unpleasant side. Ill-dressed, and untidy in her habits, absorbed in her scientific work, yet exacting the warmest expressions of affection from those whom she professed to love, Kovalevsky must have recognised his difficulties.

On the death of her father, a few years later, the relations of Madame Kovalevsky and her husband became more intimate. They settled in St. Petersburg, and for a short time all went well. Sonia contributed to newspapers, wrote poetry and dramatic criticism. Kovalevsky published several scientific works, in which field they assiduously worked together. Their first and only child was born in 1878. But the end of their married life was rapidly approaching. Some jealous suspicions on the wife's part seemed to have brought about a complete rupture, and, taking her little daughter with her, Sonia left her husband for ever, went to Paris, and there continued her studies. That she was not quite inconsolable certain particulars in the Duchessa di Cajanello's account of her friend lead us to surmise. It was whilst she was studying mathematics in Paris with a young Pole, a worker like herself, that news reached Sonia of her husband's suicide, consequent on a long series of speculative failures. But despite her morbid tendencies of thought, her scientific work was vigorously hopeful, otherwise it never could have been crowned with the triumphs which one after the other were rained upon her. In 1874 she received the degree of D.Ph. at the University of Gottingen. The French Academy presented to her the Prix Bordin, the greatest scientific distinction ever bestowed on a woman, but also one of the highest to which any mathematician could aspire; and, through virtue of her superiority over all other candidates for the post, she was appointed Professor of Mathematics at Stockholm in 1884. She made in science a great, and in general literature a considerable name, in spite of the shortness of her life. Sonia Kovalevsky's end came suddenly, one short year after her nomination to the Professorship.

But lives are to be judged as to their length by their fruitfulness. Looked at in this way, the life of Sonia Kovalevsky, as her biographer suggests, was longer than most people's; she had lived intensely, drunk deep draughts of the wells of joy and grief and of the treasures of science, and had reached heights to which only imagination can lift one.

The Duchessa di Cajanello has done her task of biographer in an admirable manner. She has shown the woman she describes as that woman would have us know her—less as the genius whose brilliant attainments placed her on a pedestal above her fellow creatures than as the woman whose mightiest effort was expended in a fruitless search for the attainment of a perfect love.

* "Sonia Kovalevsky." A Memoir by the Duchessa di Cajanello. (Walter Scott, Ltd. London. 1895.)

### A Swiss Hospital.

By a Trained Nurse.

Of the many pleasant spots which form a halting-place and a centre from whence the tourist can make excursions into the region of the boldest and most beautiful scenery in Switzerland, few are more pleasant or more advantageous as head-quarters than the charming town of Interlaken. Its name explains its situation, and those who have been there and climbed up the wood-covered hill which runs behind the town, high enough to command a panoramic view of the surroundings, will remember the lovely *coup d'œil* afforded by the town lying peacefully below, linking together the shining waters of Lake Thun and Lake Brienz, while on the other side the snowy heights of the sublime Jungfrau keep silent watch over the valley stretching at its feet.

Everywhere the eye turns it rests with delight upon some fair scene ever changing into fresh beauty as the shifting lights and shades play over its surface.

The town seems to consist chiefly of modern and palatial hotels, whose white *façades* look wonderfully clean to eyes accustomed to the all-pervading grime of our smoky England.

A wide boulevard runs through the centre of the town, with a double row of trees, beneath whose shade the shops, crowded with photos, carvings, and exquisite lace (the product of busy fingers working in the long winter months when the visitors are gone), tempt the passers-by to carry away souvenirs of their visit.

But leaving the new part of the town, we wander on down the wide road, attracted by the sight of the quaint red church spire which rises up before us out of the clustering trees, and the sight of the old building carries the mind back from the busy stir of the present day to the days long past, when a rich and famous monastery stood for many generations on this spot, until the sweeping changes of the Reformation scattered its inmates far and wide, and left the buildings standing bare ready for other uses in the changed times.

And to many uses are the old buildings now put, for in the chancel of the old church the English Church services are held, while (entering by another door) the Roman Catholics hold their services in the nave, and the Presbyterians have secured possession of the transepts for their simpler form of worship. Thus the quaint red roof draws beneath its kindly shelter men of many nations and of many creeds, resembling *en miniature* the blue sky which bends over the great human family and shelters all alike.

Passing on to the outbuildings of the old monastery, we see written over a plain door the word "Krankenhaus." A desire to compare the interior of this unpretentious institution with the hospitals we know in London prompts us to pull the iron handle of the bell, and the ring is soon answered by a deaconess, who kindly responds to our wish to see the hospital, and we step into the brick-paved entrance. Through a door opposite we catch sight of the kitchen, where two men are peeling potatoes. We conclude they are convalescent patients, but the sister says, "No, 'Gefangene'" (prisoners), which sounds odd until we remember that another part of the monastery is used as a kind of police-station, and we suppose that the services of some of the prisoners are placed at the disposal of the hospital to help in the rougher work; but the problem of how this system would work if introduced into an English hospital much exercises our mind as we go upstairs to see the wards.

The theatre at the head of the stairs is small, and plainly fitted up. The walls are hung with framed texts of Scripture, printed in plain German character.

A verandah runs round one side of the building, from which open one or two isolation rooms, in which cases of diphtheria are being nursed.

The sister showed us a large and pleasant-looking room, where paying patients are received, and it not unfrequently happens that if one of the many visitors who throng the hotels is taken ill, the doctor is glad to send his patient to the quiet shelter and kind care of the sisters at the hospital. "An English lady was here last Christmas," we are told.

We see the sitting-room for the sisters, and admire a dish of water lilies on the centre of the table. Two of the nurses had gone out early that morning before going on duty to a little lake lying in the woods, and had brought back these fair spoils. The happy faces and cheerful looks of the deaconesses are a pleasant memory as we leave the picturesque building so rich in associations, a silent witness through many generations of the old order gradually changing to yield place to the new as we see it to-day.

### Where to Go.

Queen's Hall, Langham Place.—Miss Annie Rose (Mrs. Horace Neville) intends giving a matinée in the large hall during the first week in March, of which the proceeds are to be devoted to St. Saviour's Hospital, Osnaburgh Street, N.W.

Haggerston and Hoxton District Nursing Association.—On Thursday, February 20th, the "Creation" was performed by the North-East London Choral Society at the Town Hall, Shoreditch, in aid of the funds of the Haggerston and Hoxton District Nursing Association, 105, Nichol's Square, Hackney Road, N.E. This association, which is in affiliation with the Queen Victoria's Jubilee Institute, provides trained nurses for the sick poor in their own homes, and has been doing an excellent work in a poor and crowded neighbourhood since 1889. Then there was only one nurse, now six are at work, and it is because an increase in this staff is urgently needed that a special appeal is now being made for more funds to enable the committee to add another nurse without delay. We hope the kindly effort of the North-East London Choral Society, with the aid as soloists of Miss Kate Cone, Mr. Charles Chilley, and Mr. Watkyn Mills, to help the association, will have resulted in substantial proceeds. The lady patronesses included the Marchioness of Salisbury, president of the association, and the vice-presidents, the Lady Florence Cecil and Lady Frederick Cavendish.

### Practical Points.

*A Matron, a trained nurse, writes: Is there any law making it illegal for a matron of a small infectious hospital to nurse the patients? The hospital is supported out of the rates, and can the ratepayers have a view in the matter? Also, what is the best appliance for keeping a conveyance warm whilst bringing patients to hospital?*

There is certainly no law making it illegal for the matron of a small-pox infectious hospital to nurse the patients. This type of institution is usually a small building, and, if properly organised, must be in charge of a matron who is a trained nurse of experience, the chief part of whose duty it will be to nurse patients with the aid of such assistance as the circumstances of each epidemic may demand. The ratepayers have usually a voice in the matter, as they elect the members of the sanitary authority in whose control the hospital is vested. There are various contrivances for heating ambulances, such apparatus forming a part of every well-constructed conveyance of this description. For temporary purposes an ordinary foot-warmer or hot-water bottle would prove sufficient, but for long journeys it is a matter of considerable difficulty to maintain a proper temperature by this means, and at the same time to secure efficient ventilation.

### The Position of Nurses.

Under the above title a letter is published in *The Lancet* of February 15th, which we reproduce below, as we think that the warning it contains is one which might well be taken to heart by a large number of nurses. At the same time we would point out that patients and their friends have the remedy in their own hands. When a nurse has been guilty of gossiping in the manner objected to, she should be immediately dismissed, and the cause of her dismissal made known to the institution to which she belongs, or to the doctor who recommends her. No nurse can be regarded as worthy of the position which she occupies who indulges in the objectionable practice of discussing ailments with her patients.

A correspondent in *The Lancet* of February 1st censures the loquacity of nurses from a medical point of view. May I, from a patient's point of view, ask if nothing can be done to stop nurses from regaling such patients as they may have under their care, who are able to listen to them, with minute details of the various unpleasant incidents and objects they have witnessed during operations, and of other matters which I hope I may describe without offence as "horrors of the hospital"? I happen to have had several relatives and friends under the care of trained nurses recently, and though I cannot say that their recovery was in any way retarded, I can confidently say their minds would have been easier during its progress had they been allowed to dwell upon pleasanter topics. As far as I have been able to ascertain, conversation and anecdotes of the kind indicated are indulged in almost invariably; though whether it is thought that a display of experience, and possibly callousness, will breed respect in the patient, or whether the simple pleasure of making a fellow-creature's flesh creep is their motive, as with the fat boy, I cannot tell.

### Everybody's Opinion.

[Correspondence on all subjects is invited, but we cannot in any way be responsible for the opinions expressed by our correspondents. No communications can be entertained if the name and address of the correspondent is not given, or unless one side of the paper only be written on.]

### NURSES AND LAY COMMITTEES.

"Criterion" writes: "A Superintendent of District Nurses" evidently misapprehends the meaning I intended to convey in my letter in your issue of the 25th ult. No one would say that the poor are not as much entitled to ask the services of the district nurse as the rich are to call in a trained nurse at their own expense. The district nurse exists solely for the purpose of rendering efficient help to those poor people who are unable to pay for a nurse. My complaint, however, was, of the nurse being urged by her committee to press her unsolicited services on poor people. Nearly all nurses I have met have strongly objected to this canvassing, and certainly no one likes to be told that her services are not wanted, as has been done, to my knowledge, on many occasions. My next complaint was that the nature of the disease from which the patient suffered was entered in the nurse's book, clothed in technical language certainly, but that did not prevent the committee from endeavouring to find out by what name it was known to the lay public. Now I submit that the possession of such knowledge by a committee of ladies is injurious to confidence between doctor and patients, more especially in villages and country districts, where the natural proclivity for gossip is proverbial.

### "NURSING IN CROWN COLONIES."

Mr. Walter Maguire writes: In reference to Sister Margaret's letter on the lack of nurses in British Guiana, I consider there is no British possession in the world so much neglected, and in so much need of attention by pioneers of nursing, as this colony. We have the Zenana Mission supported by thousands of energetic workers, but no serious steps have hitherto been taken towards the amelioration of the discomforts endured by the thousands of coolies on the sugar plantations. True, the estates have such a "hospital," but a native will suffer nearly any pain rather than resort to them. I have even known a man to fill an old abscess cavity in the leg with mud and turmeric, on the approach of the manager, or overseers, on their rounds of inspection; and many cases of concealed child-birth have occurred. It is also true that the estates are periodically visited by Government inspectors, but this inspection leaves much to be desired. In the first place, these "hospitals" are frequently situated ten to twenty miles from the town, difficult of access (sometimes by water only), and no resident medical man. In cases of emergency six or eight hours may elapse before the services of a qualified man can be procured, and, in fact, the trouble is seldom taken to send for him and the patient has to await his regular visit. The hospitals around Berbice and down the Corentyne Coast are raised on brick pillars 10 feet to 15 feet from the ground, a flight of wooden steps leading to a verandah, off which are situated the ward or at most two wards. The spaces between the pillars, under the hospital floor, are utilised as store-rooms, lumber-rooms, wash-house, kitchen, and "dead-house," and the flies, mosquitoes, and odours arising from this state of things are exceedingly "interesting." During my residence near Berbice some time ago a night watchman, a coolie indentured emigrant on Plantation Lochaber, was found murdered in the watch-hut on the back dam and was brought in and placed in the "dead-house." A whole week transpired before the inquiry was concluded and the order for the proper disposal of the body given. As this was during the hottest season, and, in a climate which compels burial within twelve hours of death, the discomfort to the patients in hospital during this time may be better imagined than described.

### Appointments.

Hospital for Women, West Street, Brighton.—Miss Ethel Parry has been appointed Matron of this hospital. She received her training at St. Bartholomew's Hospital, London, where she has since held the post of staff nurse. Miss Parry has our best wishes for success in her new work.

Atteryn Fever Hospital, Newport, Monmouthshire.—Mrs. Inglis has been appointed Matron of this new fever hospital. She was trained at the Victoria Infirmary, Glasgow, was afterwards charge nurse at the Newport and Monmouthshire Infirmary, and at the present moment holds the post of home sister at the Belfast Nurses' Home and Training School, in connection with the Belfast Royal Hospital. We congratulate Mrs. Inglis on her appointment.

National Hospital for the Paralysed and Epileptic, Queen's Square.—Miss R. P. Fiennes-Clinton has been appointed Lady Superintendent of this hospital. She was trained at the Leicester County Infirmary, and was for several years a sister at the London Hospital. Miss Fiennes-Clinton afterwards held the post of assistant matron at the same hospital, and has since been visitor to the nurses of the Workhouse Infirmary Nursing Association. Miss Fiennes-Clinton's appointment to the beautiful hospital in Queen's Square has given pleasure to her friends and former fellow-workers, and we wish her every success in the new work, which her varied and valuable experiences have specially fitted her to fill.

### Novelties for Nurses.

### NEW PATENT BLOTTING PAPER.

Messrs. Craig and Sons, the largest makers of blotting papers in the world, have just patented an entirely new absorbent paper which will prove a real benefit to all who use it. There certainly is nothing more aggravating than bad blotting paper, with its attendant smudging results. Messrs. Craig and Sons are to be congratulated on the successful nature of their new patent paper. It supplies a want long felt by the public. No one who has used this paper will ask for any other. It has far more absorbing power than the best blotting paper hitherto manufactured. "Spungia" will neither smudge nor spoil written matter, and will continue to absorb the ink until the paper is used up. Each sheet, made on a new process, is composed of four separate sheets, the two centres being made specially absorbent, like a sponge. Hence its name, "Spungia."

### For Reading to the Sick.

### REPOSE.

### Motto.

"Peace let us seek; to steadfast things attune calm expectations."—*Wordsworth.*

### Verses.

God for His service needeth not proud work of human skill,
They please Him best who labour most to do in peace His Will;
So let us strive to live! and to our spirits will be given
Such wings as, when our Saviour calls, shall bear us up to Heaven. —*Wordsworth.*

Put thou thy trust in God,
In duty's path go on;
Fix on His word thy steadfast eye,
So shall thy work be done.

No profit canst thou gain
By self-consuming care;
To Him commend thy cause, His ear
Attends the softest prayer.

Give to the winds thy fears;
Hope, and be undismayed.
God hears thy sighs, and counts thy tears;
God shall lift up thy head.

Through waves, and clouds, and storms,
He gently clears thy way;
Wait thou His time—thy darkest night
Shall end in brightest day. —*Martin Luther.*

### Reading.

In times of quietness the heart unfolds itself before God. If thou would'st grow in grace enter into thy closet, and shut to the door upon the world—upon that world which gets the closest to thee, and haunts thee so familiarly. Shut it, most of all, upon thy busy, unresting self, and then God shall speak to thee. It may be He will commune with thee, as He has never done before, and reveal unto thee the secret of His presence. How silent, surely, is an angel's heart when God is nigh; how, as some earthly vapour by the sun, is every power of His mighty being drawn up into adoration!

And this truly is to know Him; to be silent in His presence; to be drawn out of self, out of earthliness and the noise, and the dimness of self worship, and "to hold ourselves still in Him."—*Wilberforce.*

"The intrinsic spiritual greatness, which needed no mighty works for its external display, is evidenced by nothing more strikingly than by the Redeemer's unvarying serenity and repose. His public life was one which made continual and severe demands both on His intellectual and emotional nature; and yet, in all the most trying crises of temptation and suffering, He maintains this wonderful self-possession and tranquility undisturbed. There are, indeed, variations of feeling, alternations of joy and sorrow, flitting across His Spirit, like the shadows of clouds across a sunny landscape; but still these are no more than natural variations, the ordinary response of a strong and equal mind to the stimulus of sympathy or suffering, of success or adversity. But we never feel that, even in its most excited moments, the Spirit of Jesus is thrown off the balance; that it wastes its energy in unmeaning vehemence or unworthy complaint; that it forgets, even for an instant, its Divine purpose and Divine dignity in passionate exclamations or enraptured self-forgetfulness. . . . And yet, while the Lord is seen to be thus calm and immovable in His conscious power and impeccability, on the other hand his calmness has none of the stillness of torpidity, or the repose of stony indifference. He was as tender as strong, as humble as majestic."—*Rev. J. Moorhouse, M.A., Hulsean Lecturer.*

### The Book World for Women and Nurses.

### MAGAZINES OF THE MONTH.

The Windsor Magazine for February is full of interesting reading, the literature extending over a wide range of subjects. The contents of this month's issue include "First Impressions of the House of Commons," by Walford D. Green, M.P., a brightly-written article illustrated by photographs; "The Boyhood of Britain's Greatest Admiral," by E. Stevenson; "A Long Interruption," complete story by Mary Kernahan; and Mr. Frankfort Moore's "Through the Telescope."

Cassell's Family Magazine is worth investing in if alone for one article in the current number; but, as it happens, there are attractive features besides the exquisitely-illustrated "Backs of Cambridge Colleges." Here the drawings of these "Backs" in winter are most charmingly effected. The views are all taken in winter, with the snow on the buildings and the trees. The letterpress, by Mr. Alan St. Aubyn, is written in an easy, pleasing manner. There is a bright little story further on in the magazine by Mrs. W. K. Clifford, called "Luck for Him."

The Cornhill Magazine contains Adventure L. of Mr. Crockett's "Cleg Kelly"; and the other serial, "Clarissa Furissa," by Mr. W. E. Norris, continues to be interesting. The short story this month in these pages is called "The Consul's Wife"; and other articles are on "Impressions of a First Night" and "Our Old Town Walls."

### Notes and Queries.

### Queries.

(152) *Salary.*—Kindly tell me what salary a matron of a hospital containing 75 beds ought to receive?—*Gloucestershire.*

(153) *St. Margaret's Guild.*—Can you tell me anything about St. Margaret's Guild, which I understand is connected with the Red Cross Nurses' Association?—*J. E. B.*

(154) *Maternity Hospitals.*—Where can I get the addresses of maternity hospitals, and will you advise me as to a medical dictionary suitable for a nurse?—*Birkenhead.*

(155) *Fees.*—Can you tell me the lowest fee for which I could learn midwifery, and also the cost of uniform and inclusive expenses at Queen Charlotte's?—*Constant Reader.*

(156) *Training in Germany.*—Can you recommend me a good general hospital in North Germany where probationers are trained and given a certificate, and also receive a salary?—*Probationer.*

(157) *Uniform.*—How soon after entering a hospital as probationer can I wear the outdoor uniform?—*Probationer.*

(158) *Home for Inebriates.*—Can you kindly tell me of a home for a well-educated middle-class man of inebriate habits, where by strict discipline he may be helped to overcome this pernicious failing? He would not be able to pay very much.—*Sister Grace.*

(159) *Queries.*—Thank you for your kind information in this column. Please let me know if there is a fee to pay.—*C. S.*

### Answers.

(152) *Salary* (*Gloucestershire*).—Much depends upon the duties required of the matron, and on other details not mentioned in your letter. Speaking broadly, the matron of a hospital of 75 beds would usually be found to receive a salary varying between £40 and £80.

(153) *St. Margaret's Guild* (*J. E. B.*)—The Red Cross Nursing Sisters' Training School for Nurses is an Irish institution. You had better apply to the Secretary, 87, Harcourt Street, Dublin, for the information you require.

(154) *Maternity Hospitals* (*Birkenhead*).—(1) You will find a complete list, with particulars, in "How to Become a Nurse," Scientific Press, 428, Strand. (2) "The Nurse's Dictionary," by Honnor Morten, Scientific Press, price 2s., is compiled expressly for nurses; or more expensive works are Quain's "Dictionary of Medicine," price £1 14s., or Hoblyn's "Dictionary of Medical Terms," price 10s. 6d. These can sometimes be procured second hand.

(155) *Fees* (*Constant Reader*).—Write to the matron of Queen Charlotte's Hospital, Marylebone Road, for the particulars you desire. You can get the addresses of all the lying-in hospitals from "Burdett's Hospital Annual." Wherever you go you should make up your mind to remain for the full term of training, and get the certificate of the London Obstetrical Society.

(156) *Training in Germany* (*Probationer*).—If you will send further particulars as to age, training already received, and if your intention is to continue nursing in Germany in hospital or private work, we may be able to help you.

(157) *Uniform* (*Probationer*).—Any woman who enters a hospital for training as a nurse can wear an outdoor uniform if it suits her so to do, and if it is the rule of the hospital for the staff to wear it she will be required to provide herself with the necessary cloak and bonnet when she has passed her month's probation. Few hospitals provide outdoor uniform, and it is not always obligatory on the nursing staff to wear it.

(158) *Home for Inebriates* (*Sister Grace*).—Write to Dr. Anderson, Dalrymple Home, Ricksmanworth, for particulars of admission. Perhaps if the terms there are beyond the patient's means Dr. Anderson may be able to recommend to you some other home.

(159) *Queries* (*C. S.*)—There is no fee to pay for information given in "Notes and Queries." We are always very glad to be of any help to nurses in this way. It is a true pleasure to feel that any trouble taken in reply to inquirers is valued and appreciated by our readers.

## Figures and Tables

**Figure f1:**